# Making deep‐tissue monitoring clinically accessible with soft, biodegradable inductor–capacitor implants

**DOI:** 10.1002/ctm2.70844

**Published:** 2026-09-18

**Authors:** Yuqun Lan, Shuang Li, Yewang Su

**Affiliations:** ^1^ State Key Laboratory of Nonlinear Mechanics Institute of Mechanics Chinese Academy of Sciences Beijing China; ^2^ School of Engineering Science University of Chinese Academy of Sciences Beijing China; ^3^ Department of Engineering Mechanics Institute of Biomechanics and Medical Engineering Tsinghua University Beijing China; ^4^ Beijing Key Laboratory of Engineered Construction and Mechanobiology Institute of Mechanics Chinese Academy of Sciences Beijing China

1

Physiological information obtained from deep tissues is essential for precision medicine, as it aids in postoperative assessment and enables timely diagnosis and treatment. For instance, elevated intracranial pressure after surgery may signal cerebral oedema or reduced compliance, while rising intra‐abdominal pressure can impair perfusion or warn of compartment syndrome. Shifts in temperature or pH may indicate infection or leakage, and abnormal tissue strain can expose mechanical dysfunction. These local changes can precede overt systemic symptoms and influence whether clinicians intensify surveillance or initiate an intervention. Yet current clinical tools provide only intermittent or indirect access to such information. Medical imaging, including magnetic resonance imaging (MRI), computed tomography and ultrasound, provides spatially resolved information on anatomy, lesion location and tissue structure, as well as metabolic activity or derived functional measures in selected modalities (Figure 1a). Although indispensable for diagnosis and treatment planning, most imaging examinations depend on specialised equipment and provide episodic snapshots rather than continuous measurements. In routine clinical practice, blood tests and laboratory analyses offer valuable systemic biomarkers. However, these methods are intermittent and lack the ability to continuously capture long‐term, localised changes occurring inside deep organs or at surgical sites. Continuous access to such local physiology therefore remains an important unmet clinical need.

**FIGURE 1:**
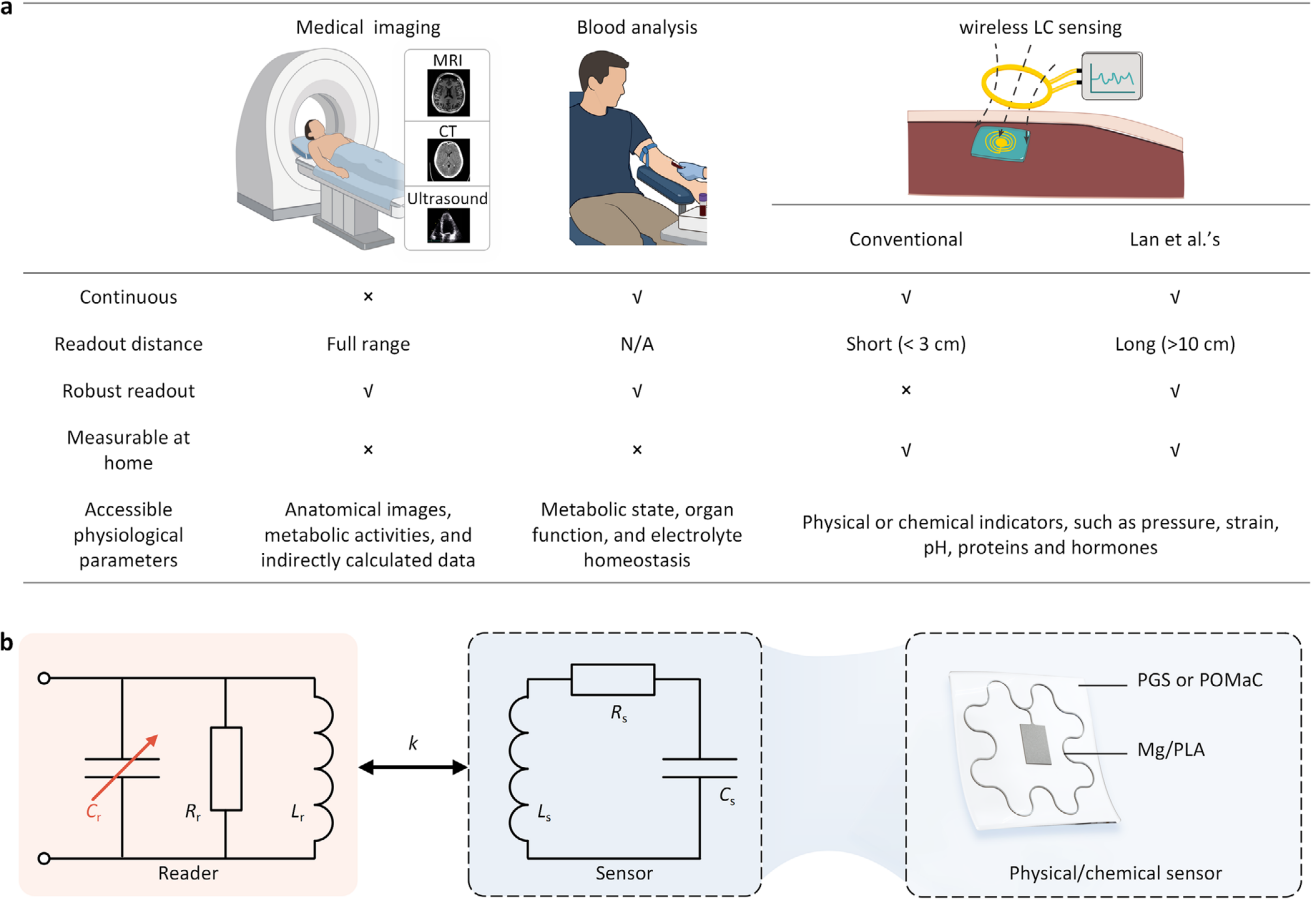
Description of methods for obtaining physiological information from deep tissues. (a) Comparison of different methods. (b) Demonstration of Lan et al.’s wireless inductor–capacitor (LC) sensing (R: resistance; C: capacitance; L: inductance; subscript ‘r’ represents reader, ‘s’ represents sensor).

Soft wireless implants offer a new route to continuously monitor deep physiological information. Passive inductor–capacitor (LC) sensors are particularly attractive for temporary implantation because they do not require an implanted battery. Pressure, temperature, strain or local chemical environment changes the resonant frequency of LC sensors, and an external reader can detect this frequency. The simple passive architecture of LC sensors is compatible with flexible and bioresorbable materials, enabling the implants to be designed to disappear after the clinically relevant monitoring period. First demonstrated in a pressure‐sensitive telemetering capsule in 1957, this basic readout architecture remains widely used in contemporary passive and bioresorbable implants.^1^, ^2^, ^3^ The principal limitation, however, is not the ability to sense a physiological variable, but the ability to read it reliably through deep tissue. Conventional LC readers generally operate over only a few centimetres.^2^, ^4^ Recently developed parity–time symmetric and exceptional‐point readout systems can amplify weak resonance changes and extend the detectable range,^5^, ^6^ but their accuracy remains sensitive to the strength of the wireless interaction between the implant and reader. In patients, breathing, posture, tissue thickness and ordinary movement continuously alter the strength of the wireless interaction, leading to incorrect measurement results. A clinically useful passive implant must therefore maintain accurate readout, rather than merely produce a detectable signal under fixed laboratory conditions.

Against this background, Lan et al. proposed a soft, biodegradable, wireless sensing platform combining a pole‐moving sweeping readout with an integrated folded structure^4^ (Figure 1b). During frequency sweeping, the resonant frequency of the reader is synchronously adjusted, producing a recognisable phase response near the resonant frequency of the sensor even when the coupling changes with distance or angle. The integrated folded structure addresses a complementary challenge by combining mechanical flexibility with electromagnetic function, enabling a soft, integrally biodegradable LC circuit to retain the inductance required for long‐distance wireless communication. Because the platform senses changes in resonant frequency, it can in principle detect a wide range of physical, chemical, and biological signals, as long as the target stimulus can induce a measurable change in the dielectric properties or volume of an appropriately designed sensing material, thereby altering the capacitance and resonant frequency. Multiple biomedical demonstrations were designed to examine different aspects of clinically relevant environments. For example, monitoring the strain of an ex vivo pig heart tested whether the platform could capture continuous and consistent signals from a moving and deformable organ under mechanical loading. In vivo pressure and temperature monitoring inside the abdominal cavity of a horse demonstrated deep‐tissue readout in a large animal. Together, these experiments show that the platform can maintain accurate sensing without strict positional control.

The clinical importance of this platform lies in its potential to complement medical imaging by providing continuous and accurate access to physiological signals deep within the body. Several future clinical scenarios illustrate this opportunity. After major abdominal surgery, rising intra‐abdominal pressure can indicate infection, bleeding, pancreatitis or evolving abdominal compartment syndrome. Such measurements could potentially inform fluid management, ventilator settings and decisions regarding further intervention. Local temperature trends may provide an additional indicator of organ inflammation.^4^ Local temperature changes around a kidney graft may precede conventional blood markers of rejection and could support earlier adjustment of immunosuppression.^7^ In gastric surgery, a local decrease in intra‐abdominal pH could provide an early warning of gastric leakage before symptomatic deterioration, creating an opportunity for earlier imaging, drainage or reoperation.^3^ After neurosurgical procedures or traumatic brain injury, rising intracranial pressure can indicate intracranial hypertension and declining compensatory reserve, increasing the risk of secondary brain injury and prompting timely treatment. Brain temperature may reflect fever, inflammation or metabolic stress, and cannot always be inferred reliably from body‐surface temperature. Continuous monitoring of these two variables could therefore provide an earlier warning between imaging examinations and support decisions about repeat imaging or treatment escalation.^8^, ^9^

Moving this concept towards clinical use will require progress along four linked paths (Figure 2). First, human feasibility studies should evaluate not only biocompatibility and signal stability, but also agreement with established clinical measurements and the ability of the data to change management in predefined indications. Second, calibration must be treated as a time‐dependent property. Material degradation can alter the geometry, dielectric environment and coupling of the implant. Future devices may therefore require a reference resonator, a second measurement channel or model‐based recalibration to distinguish physiological changes from device drift. Third, the reader should evolve into a portable bedside system that automatically tracks and compensates for changes in its resonance frequency. This system converts resonance shifts into clinically interpretable parameters and trends that can be incorporated into routine patient monitoring. Finally, translation will require systematic evaluation of sterilization, MRI compatibility, degradation products and device failure modes, together with a regulatory strategy that considers the implant, reader, implantation procedure and degradation process as one integrated platform.

**FIGURE 2:**
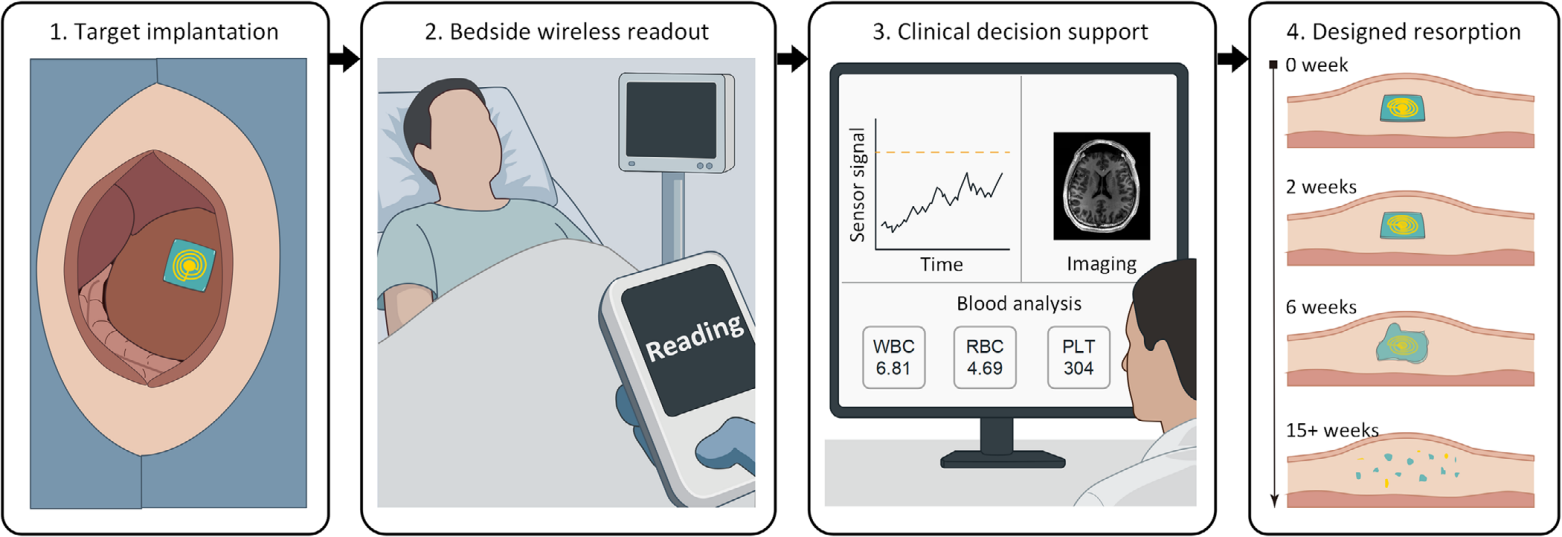
Towards clinical translation of soft biodegradable wireless inductor–capacitor (LC) implants for deep‐tissue monitoring.

## AUTHOR CONTRIBUTIONS

Yuqun Lan: Conceptualization, Writing – Original Draft, Visualization. Shuang Li: Conceptualization, Writing – Review & Editing. Yewang Su: Conceptualization, Writing – Review & Editing.

## ETHICS STATEMENT

Ethics approval was not required for this article, as it discusses previously published research and reports no new studies involving human participants or animals.

## CONFLICT OF INTEREST STATEMENT

The authors declare no conflict of interest.
